# Comparison of the Clinical Characteristics of Histiocytic Sarcoma in Bernese Mountain Dogs and Flat-Coated Retrievers

**DOI:** 10.3390/vetsci9090498

**Published:** 2022-09-11

**Authors:** Suzanne A. Erich, Jane M. Dobson, Erik Teske

**Affiliations:** 1 Department of Clinical Sciences, Faculty of Veterinary Medicine, Utrecht University, P.O. Box 80154, 3508TD Utrecht, The Netherlands; 2 Department of Veterinary Medicine, University of Cambridge, Cambridge CB3 0ES, UK

**Keywords:** histiocytic sarcoma, Bernese Mountain Dog, Flat-Coated Retriever, clinical characteristics, pathology

## Abstract

**Simple Summary:**

Histiocytic sarcoma (HS) is a malignant hematopoietic tumor. It can affect any organ in the body and, therefore, can have a broad spectrum of clinical presentations. So far, no complete overview exists of the array of clinical aspects of HS in specific dog breeds in large groups. Therefore, we investigated the clinical characteristics of HS in a population of Bernese Mountain Dogs (BMD; n = 365) and Flat-Coated Retrievers (FCR; n = 289), which are two of the most affected dog breeds. The dogs were selected from veterinary pathology services, and each dog’s clinical and diagnostic characteristics were retrospectively collected. Localized HS was reported significantly more frequently in the FCR (60.6%) than in the BMD (39.2%), and disseminated HS was recorded significantly more frequently in the BMD (60.8%) than in the FCR (39.4%). Lameness as a clinical symptom was observed more frequently in the FCR, predominant in the front legs. With blood examination, the BMD had significantly more often leukocytosis and thrombocytopenia, while no difference in the occurrence of anemia was observed. Strikingly hypercalcemia was only observed in 15 BMD and in none of the FCR in which blood examination was performed. The new information provided in this study can aid the diagnostic process and allow for prompt treatment recommendations.

**Abstract:**

Histiocytic sarcoma (HS) is an aggressive malignant tumor of histiocytes, which can affect almost any organ in the body and is characterized by a broad array of tumor locations and clinical presentations. So far, no complete overview exists of the array of clinical aspects of HS in specific dog breeds in large groups. Therefore, we investigated the clinical characteristics of HS in a population of Bernese Mountain Dogs (BMD; n = 365) and Flat-Coated Retrievers (FCR; n = 289), which are two of the most affected dog breeds. Cases were selected from databases from different pathology services, and clinical information was retrospectively collected for each case. Localized HS was reported significantly more frequently in the FCR (60.6%) than in the BMD (39.2%), and disseminated HS was recorded significantly more frequently in the BMD (60.8%) than in the FCR (39.4%). Lameness was seen more often in FCR than in BMD, and the vast majority (78.1%) of LHS leading to lameness was located in the front legs in the FCR, while in the BMD, there was a more even distribution. BMD had significantly more often leukocytosis and thrombocytopenia, even corrected for the type of HS, than FCR. No significant difference in the frequency of anemia was recorded between BMD and FCR. In those dogs in which blood examination was performed, hypercalcemia was diagnosed in 15 BMD, while none of the FCR had hypercalcemia. The new information provided in this study can aid the diagnostic process and allow for prompt treatment recommendations.

## 1. Introduction

Histiocytic sarcoma (HS) is aggressive neoplasia of histiocytic cells, which includes the dendritic cells and macrophages [1,2]. Histiocytic cells can be found throughout the entire body, but some have preferential organs [1]. In HS, a single mass can be found (localized HS), or it can be widespread throughout one or several organs (disseminated HS) [1]. It seems that dendritic cells are the cells of origin in most cases of HS, but it is not exactly known which precise dendritic lineages can be affected [1], and in addition, a haemophagocytic variant from macrophages has also been recognized [2].

A high incidence is seen in several dog breeds, especially in Bernese Mountain Dogs (BMD) [1,2] and Flat-Coated Retrievers (FCR) [1,3,4,5,6,7]. Recent studies have identified HS-associated loci on specific chromosomes in both BMD and FCR [8,9,10]. If untreated, the disease is usually fatal. A chemotherapy median response rate of up to 58% [11] and median survival times of 185 days have been reported for systemic HS [12], and for localized HS, combination therapy of surgery and chemotherapy resulted in median survival times of 568 days [13].

In an earlier study, it was reported that BMD and FCR had a very high percentage of death due to HS, 15.3% and 14.3%, respectively [14]. In this study, there was a significant difference in median age at which the disease was diagnosed, 6.3 years in BMD and 8.8 years in FCR. Apart from some case series and some studies without cytological or pathological confirmation of the diagnosis of all cases [2,15,16,17,18], large studies on clinical aspects of these two breeds and the differences between them are missing. When only the more common presentations are known and the less common ones are missed, this can delay the diagnostic process and can reduce the survival rate. 

The purpose of this study is to investigate the clinical characteristics of HS in a large population of dogs of the breeds most often affected by HS: Bernese Mountain Dogs (BMD) and Flat-Coated Retrievers (FCR). The study is based on the current knowledge of HS in dogs and humans and evaluates the diagnostic investigations which can be made workable for routine implementation, including cytology, histopathology, and immunohistochemical markers. The study provides an overview of the scope of possible clinical presentations, including clinical signs, possible affected organs, and hematological and biochemical abnormalities and the differences in these parameters between both breeds. A workable organ distribution is presented, which encompasses the whole range of locations affected by HS, which can support the diagnostic and prognostic counseling process of the veterinarian.

## 2. Materials and Methods

### 2.1. Case Selection 

Cases for potential inclusion in the study were BMD and FCR with a confirmed histological and/or cytological diagnosis of MH/HS. Cases were collected from the Netherlands, Germany, Belgium, Switzerland, and the United Kingdom (FCR only), using database searches for the diseases listed above in the Veterinary Pathological Diagnostic Centre (VPDC) and the University Veterinary Diagnostic Laboratory (UVDL) of the University of Utrecht (NL), Veterinary Specialist Centre The Wagenrenk (Wageningen, NL), Valuepath (Hoensbroek, NL), Vetipath (Westervoort, NL), and the Veterinary Pathology Service of the University of Cambridge (UK). The start of the selection period was dependent on the start of digitalization of the database of each laboratory which varied from 1989 to 2004 and took place until July 2011. Veterinary records of the candidates were collected from the associated veterinarian. Dogs were only included in this study when being family-owned purebred BMD or FCR with complete clinical and pathological reports and associated samples available for review. Pedigree data were collected from the owner and verified in the digital databases of BMD and FCR of the Dutch Kennel Club (Amsterdam, the Netherlands). All BMD and FCR cases’ age, sex, history, clinical signs, tumor locations, and pathology and/or cytology reports were collected. In cases of doubt on the cytology report, slides were re-evaluated (ET). In addition, if present, results of diagnostic imaging and laboratory results were also collected.

### 2.2. Diagnosis

The diagnosis was made based on cytology or histology with immunohistochemistry, as reported before [19]. Localized HS was defined as single or multiple tumors in a single organ with or without involvement of its regional lymph nodes. Disseminated HS was defined as the presence of tumors in multiple organs and/or involvement beyond the regional lymph nodes.

### 2.3. Statistics

Data were entered in an EXCEL spreadsheet and analyzed with SPSS 25.0. (IBM SPSS Statistics for Windows, Armonk, NY, USA). After testing for normality with the Kolmogorov–Smirnov test, *t*-test and ANOVA were used to test for significant differences in characteristics between BMD and FCR. The chi-squared-test likelihood ratio was used to test associations between categorical data. *p* < 0.05 was considered as significant.

## 3. Results

### 3.1. Demographics

A total of 654 dogs (365 BMD and 289 FCR) were diagnosed with HS by cytology and/or pathology with immunohistochemistry. In 143 dogs (44 FCR and 99 BMD), the diagnosis was made by cytology only, in 282 dogs (144 FCR and 138 BMD) by histopathology only, and in 228 dogs (101 FCR and 127 BMD) by both cytology and histopathology. Total post-mortem examinations were only performed on 58 dogs (43 BMD and 15 FCR). The descriptives of these dogs are presented in Table 1. The age at diagnosis was significantly different between the two breeds (*p* < 0.001). The mean age of the 365 BMD cases was 6.6 years (range 0.6–12.2 years), while the mean age of the 289 FCR cases was 8.2 years (range 0.6–13.7 years).

Of the 365 BMD cases, 181 were male and 184 were female (M:F = 1:1.02), of which 41.4% and 62.0% were neutered, respectively. Of the 289 FCR cases, 142 were male and 147 were female (M:F = 1:1.04), of which 27.5% and 47.6% were neutered, respectively. Significantly fewer FCR were neutered compared to the BMD, both male and female dogs (*p* < 0.001) (Table 1).

Localized HS was diagnosed in 318 dogs (175 FCR vs. 143 BMD) and disseminated HS in 336 dogs (114 FCR vs. 222 BMD). Localized HS was reported significantly (*p* < 0.001) more frequently in the FCR (60.6%) than in the BMD (39.2%), and disseminated HS was reported significantly (*p* < 0.001) more frequently in the BMD (60.8%) than in the FCR (39.4%).

### 3.2. Presenting Signs

The main clinical signs are summarized in Table 2. Systemic signs such as pale mucous membranes, weight loss, anorexia, icterus, pyrexia, and lethargy were seen significantly more often in BMD than in FCR. In contrast, lameness was significantly more frequently reported in FCR than in BMD.

In relation to the type of HS (localized vs. disseminated), significant differences in clinical signs were found. Pale mucous membranes (*p* < 0.001), icterus (*p* < 0.001), pyrexia (*p* < 0.001), lethargy (*p* < 0.001), weight loss (*p* < 0.001), anorexia (*p* < 0.001), dyspnoea (*p* = 0.001), coughing (*p* = 0.001), polydipsia (*p* = 0.023), diarrhea (*p* = 0.024), and ascites (*p* < 0.001) were recorded significantly more often in cases with disseminated HS than in localized HS. Lameness was more often recorded in localized HS than in disseminated HS (*p* < 0.001).

Several different neurological signs were reported. Paresis/paralysis in 15 dogs (8 BMD, 7 FCR), ataxia in 7 dogs (6 BMD, 1 FCR), nystagmus only in 3 BMD, incontinence in 2 BMD, Schiff–Sherrington phenomenon and Horner syndrome in 1 BMD, respectively.

Other less frequent clinical signs reported by the owner were melena, adipsia, retching, dysphagia or change in barking sound, increased swallowing or licking of lips, throat scraping, haematuria, and problems with voiding or defecation. Other less frequent clinical signs were edema/engorgement, pain, eye problems, cyanosis, epistaxis, delayed wound healing, panting, changed respiration, stridor, hydrothorax, sneezing, and a swollen nose. 

### 3.3. Blood Results

Blood work-up at the time of diagnosis was performed in 196 BMD (53.7% of all BMD) and 87 FCR (30.1% of all FCR) (Table 3). A significantly (*p* < 0.05) larger proportion of BMD had blood examination performed than FCR. Anemia was the most common hematological abnormality, observed in 76.5% of all cases in which blood examination was performed. No significant differences between breeds were recorded regarding the packed cell volume (PCV). In most cases, the anemia was regenerative based on reticulocyte count, and this percentage in BMD and FCR was equal (74.1% vs. 70.0%). A PCV below 0.30 l/l was more often seen in DHS than in LHS cases (*p* < 0.001).

A full blood count was conducted in 108 BMD and 44 FCR. Leukocytosis was more often reported in the BMD (*p* < 0.001), and the median value was also higher in the BMD (20.8 vs. 13.5 × 10^9^/L), while no significant differences were found between LHS and DHS cases. Monocytosis, neutrophilia, and lymphopenia were regularly seen in both breeds, but no significant differences were present. In 3 BMD (2.8%), malignant histiocytic cells were reported in the peripheral blood smear. Thrombocytopenia was more often recorded in BMD than in FCR (*p* = 0.027), and the median value was also lower (76 vs. 120 × 10^9^/L), reflecting the fact that thrombocytopenia was more often present in DHS cases (*p* = 0.003). The Coombs test was positive in 1/18 BMD and 2/5 FCR. Coagulation was prolonged in 8/17 BMD and 4/5 FCR. 

Hypoalbuminemia was seen in an equal percentage of BMD and FCR and also in LHS and DHS, with a median value of 22 g/L, and no significant relation with involvement of the liver was found (*p* = 0.658). Hypercalcemia was only present in the BMD (*p* < 0.001), and there was no significant difference in LHS and DHS cases (*p*= 0.733). Alkaline phosphatase (AP) was elevated in an equal percentage of BMD and FCR, and there was no significant difference in LHS and DHS cases (*p*= 0.215).

### 3.4. Imaging

Imaging techniques (radiography, ultrasound, CT, MRI, endoscopy, myelography) were significantly (*p* < 0.001) more often applied in BMD than in FCR (70.4% vs. 54.3%, respectively). While in FCR, significantly (*p* < 0.001) more often imaging of an extremity was performed (30.1% vs. 11.2% in BMD). Imaging of the thorax and/or abdomen was performed more often in BMD (63.6% vs. 36.6% in FCR).

### 3.5. Location of Tumors

**Figure 1:**
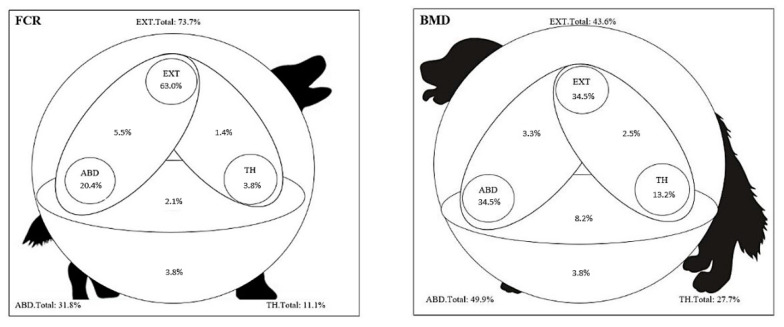
Involvement of the body compartments in HS in the FCR (**left**) and BMD (**right**). The distribution is significantly different between these two breeds (*p* < 0.001). EXT: any organ external to abdomen/thorax; ABD: abdominal; TH: thoracic.

The distribution of HS at diagnosis over the specific organs is displayed in Table 4 for FCR vs. BMD. Location is based on clinical examination for all dogs, and for a subgroup on imaging, explorative laparotomy, and p.m. examination of dogs that were euthanized at diagnosis. Rare tumor locations were bone marrow (FCR 2×, BMD 5×), testicle/scrotum (FCR 1×, BMD 4×), prostate (BMD 4×), adrenal (BMD 2×), gallbladder (BMD 1×), and vagina (BMD 1×). (In the majority of HS cases, a single organ was involved (BMD 54.2%; FCR 62.6%), with only one single mass more frequent in FCR (79.6%) than in BMD (61.6%). For all HS dogs, a single tumor mass was found more often in FCR than in BMD (53.3% vs. 36.7%; *p* < 0.001). The distribution of the tumor masses over the body compartments ‘external’, ‘abdomen’, and ‘thorax’ is displayed in Figure 1, based on physical examination and staging to the extent that the latter has been done.

#### 3.5.1. Abdomen

On ultrasound, the abnormal appearance of the spleen and liver was significantly more often detected in the BMD than in the FCR (*p* < 0.001). In the BMD, significantly more diffuse patterns were seen in the spleen and liver than in the FCR (*p* < 0.001). In cases with LHS, only single masses were seen in the liver. Involvement of the abdominal or pelvic lymph nodes was with single nodes (BMD 61.7%; FCR 44.0%) or multiple nodes (BMD 38.3%; FCR 56.0%). Involvement of the spleen, liver, lymph nodes, and kidneys were all significantly more frequently found in DHS than in LHS (*p* < 0.001). Rare locations were the stomach wall (3 BMD and 1 FCR), intestinal wall (2 FCR), adrenal glands (2 BMD), gallbladder (1 BMD), prostate (1 BMD), and vaginal wall (1 BMD).

#### 3.5.2. Thorax

Radiographically, involvement of the lungs and thoracic lymph nodes was more often recorded in the BMD than in the FCR (*p* < 0.001), and this was sometimes accompanied by pleural effusion (BMD 4.7%; FCR 16.0%). The most commonly involved thoracic lymph nodes were the tracheobronchial lymph nodes (BMD 65.0%; FCR 66.7%), followed by sternal lymph nodes (BMD 45.0%; FCR 66.7%) and mediastinal lymph nodes (BMD 27.5%; FCR 40.0%). Involvement of lungs and thoracic lymph nodes were both significantly more often reported in DHS than in LHS (*p* < 0.001).

#### 3.5.3. External

Limbs and peripheral lymph nodes were more often affected in the FCR than in the BMD (*p* <0.001 and *p* = 0.010, respectively). If peripheral lymph nodes were affected the most frequent was the prescapular (BMD 40.0%; FCR 54.9%), followed by the axillary (BMD 28.3%; FCR 31.0%), the popliteal (BMD 25.0%; FCR 23.9%), the inguinal (BMD 15.0%; FCR 5.6%), the mandibular (BMD 8.3%; FCR 2.8%) and the retropharyngeal lymph nodes (BMD 3.3%; FCR 1.4%).

Involvement of the limbs was recorded more often in FCR than in BMD (46.0% vs. 16.7%; *p* < 0.001). In FCR, front legs were significantly (*p* < 0.001) more often the primary site of the HS, while in the BMD, there was no significant difference between front and hind limbs. In both breeds, limb-HS was most often located near and/or in a joint and/or in adjacent bone: stifle (BMD 47.5%; FCR 11.5%; *p* < 0.001)), elbow (BMD 27.9%; FCR 22.9%), shoulder (BMD 4.9%; FCR 25.2%; *p* < 0.001), humerus (BMD 4.9%; FCR 22.1%; *p* < 0.001), skin (BMD 8.2%; FCR 4.6%), digit (BMD 3.3%; FCR 3.1%), carpal joint (BMD 1.6%; FCR 1.6%), femur (BMD 1.6%; FCR 4.6%), tarsal joint (BMD 1.6%; FCR 1.5%), tibia (BMD 0.0%; FCR 1.5%), hock (BMD 0.0%; FCR 0.8%), and radius (BMD 0.0%; FCR 0.8%).

No breed difference was noted for HS on the trunk (BMD 8.5%; FCR 10.0%). Most masses were single (BMD 71.0%; FCR 72.4%) and were most often located near the shoulder blades (BMD 22.6%; FCR 6.9%), pelvis (BMD 9.7%; FCR 17.2%), or groin (BMD 9.7%; FCR 20.7%).

Affected sites on the head (BMD 3.0%; FCR 4.5%) included skin (BMD 54.5%; FCR 15.4%), mucosa (BMD 18.1%; FCR 15.4%), gingiva (BMD 9.1%; FCR 0%), hard palate (BMD 9.1%; FCR 30.8%), ear (BMD 0%; FCR 23.1%), eye cavity (BMD 0%; FCR 7.7%), and epiglottis as well as soft palate, tongue, and mucocutaneous border of the lip (BMD 9.1%; FCR 0%).

## 4. Discussion

As previously reported in the literature [11], in our study, BMD were affected by HS at a significantly younger median age than FCR and other dog breeds [20,21,22,23,24,25,26]. The lower age limit in both breeds was 1.6–1.8 years; however, in both breeds, there was an exceptional young animal of 7 months of age diagnosed with HS. This young age has not been reported previously in BMD, FCR, or other dog breeds [2,14,24,27,28]. The median age of HS-FCR was comparable to the literature [4,14,29].

We found in the BMD and in the FCR an equal distribution of HS in males and females, comparable with our data collected earlier through the Kennel Clubs [14]. In the literature, male–female distribution of BMD cases varies [2,27,28,30,31]. In FCR, the variation in this distribution is smaller, with almost equal numbers of males and females affected [3,29,31]. The sex distribution varies in other studies with multiple dog breeds [20,21,22,25,32] but is equal in all studies with a somewhat larger number of cases [17,18].

The frequency of anorexia was more than 2× higher in the HS-BMD, and the frequency of anemia and lethargy was almost 2× higher in the HS-FCR. This correlates with the higher incidence of internal HS and DHS in the BMD. Pale mucous membranes in almost all our FCR are caused by anemia (95.7% of the pale FCR had a low PVC), while in 32.2% of BMD, a circulatory disorder (hypovolemia, shock, cardiac involvement) induced by HS is the cause. Interestingly we found pyrexia twice as frequently in BMD as in FCR. Pyrexia has been reported previously in BMD [15,27,28,33,34] in other/multi-breed-studies [11,23,35,36] but the frequency was very variable. It is reasonable to assume that this is a paraneoplastic syndrome related to the DHS. In FCR, pyrexia has only been reported before in one case [37].

Lameness was seen more often in FCR than in BMD and must be related to the higher incidence of periarticular LHS in that breed compared to BMD. Interestingly the vast majority (78.1%) of LHS leading to lameness was located in the front legs in the FCR, while in the BMD, there was a more even distribution. An association with prior joint disease was reported before for several breeds [38,39]; however, this association was absent in FCR.

Cough occurred with a significantly higher frequency in BMD than in FCR, which correlates with the higher incidence of thoracic HS in BMD. Cough and dyspnoea have been reported in several smaller studies in BMD [15,27] and in studies with multiple breeds [11,17,21,23] with a somewhat higher frequency. However, this was directly related to the selection of a higher proportion of thoracic HS cases. In FCR, cough and dyspnoea have not been reported before as separate signs.

Paresis/paralysis has been reported before in BMD [2,16,28,40] and in other/multi-breed-studies [22,25,36,41,42,43,44,45,46,47], however, only once before in one FCR case [48]. Schiff–Sherrington phenomenon and Horner syndrome has not been reported before in any breed with HS. Nystagmus has only been reported before in one Miniature Schnauzer with HS [49].

Although no significant difference was seen in anemia cases, BMD had a significantly more frequent occurrence of leukocytosis and thrombocytopenia, even corrected for the type of HS than FCR. This difference in leukocytosis and thrombocytopenia between BMD and FCR has not been reported before. In BMD, tumor tissue of HS has a more inflammatory component than in FCR, including both lymphocytes and neutrophils [19], which could be associated with peripheral leukocytosis. Leukocytosis is a well-known paraneoplastic syndrome in dogs, in which expression of G-CSF and GN-CSF, as well as IL-6, has been reported to be increased [50]. Chronically activated leukocytes supply several growth factors, stimulating cancer and stromal cells, and are associated with a poor prognosis [51]. It will be interesting to see in future studies if this is also associated with prognosis in HS.

In contrast to our study in which the anemia was regenerative in more than 70% of the cases in both BMD and FCR, in the majority of BMD HS cases reported in the literature, anemia has been non-regenerative [2,33,36,52,53,54]. In FCR, regenerative anemia was also only reported before in two case reports [37,55]. In the majority of other/multi-breed studies, anemia was non-regenerative. Based on the data of our study, we cannot explain this discrepancy with the literature.

In both BMD and FCR, the percentage of dogs with hypoalbuminemia was relatively high. In the majority of reports about hypoalbuminemia in other/multi-breed studies, the percentage of affected cases was lower than in BMD and FCR [11,13,17,21,23,56,57,58,59]. Albumin is a negative acute phase protein, and hypoalbuminemia has been associated with inflammation, cancer, and trauma [60]. Increased IL-6 levels were found in dogs with HHS leading to suppression of albumin synthesis in the liver [37]. It has been suggested that the level of hypoalbuminemia can be a potential help in the differentiation of HS and HHS [30]. The reason for the higher percentage of hypoalbuminemia in BMD and FCR remains unclear.

Of interest is the fact that hypercalcemia was only observed in 15 BMD and in none of the FCR in which blood examination was performed. Although total calcium was measured, it was corrected for albumin/protein concentrations. No other abnormalities were found in the hypercalcemic dogs that could explain the hypercalcemia except for the tumor. So far, hypercalcemia has only been reported in one dog with histiocytosis [61] but not in the BMD. In our study, no significant differences in the type of HS, LHS, or DHS were found in the hypercalcemic dogs. Therefore, the larger proportion of LHS in FCR is not likely an explanation for the absence of hypercalcemia in this breed.

Imaging can play an important role for both diagnostic and staging purposes in dogs with HS. On thoracic radiographs, we found a diverse involvement of lung lobes by HS in BMD and FCR. In contrast to Barret et al. [62], the right cardiac/middle lung lobe was more frequently involved. As previously reported [34], both single and multiple masses can be seen in the lungs, as also consolidation of lung lobes and enlarged lymph nodes. Ultrasound of the abdomen, especially in BMD, is an important imaging method to detect HS tumors affecting the viscera. Although the ultrasonographic patterns are not specific for HS [56,63], because of the high prevalence of HS in BMD and FCR, there is a high probability of HS, especially when multiple nodes are seen in the spleen [64] and/or liver [65].

This is the first report of the LHS: DHS distribution in BMD and FCR with a large number of HS cases. Interestingly the distributions are exactly reversed in BMD (more DHS) and FCR (more LHS). LHS:DHS distributions in BMD described before are comparable [1] or completely different [17,24]. Those in FCR described before are comparable [1] or less pronounced [17]. In other/multi-breed studies, an almost equal distribution of LHS and DHS is seen [1] or a distribution comparable to that in BMD [23] or almost comparable to that in FCR [21].

The distribution of HS lesions can also be described by the distribution within the three body compartments, ‘external’, ‘abdomen’, and ‘thorax’. This is the first time this distribution has been used, and it clearly makes a distinction between the distribution of lesions in BMD and FCR (Figure 1) and could be useful for the veterinarian, specialist diagnostic imaging, and researcher and for defining subtypes. For example, the veterinarian, in consultation with the owner, can choose the most suitable compartment for further examination with imaging. Regarding the possible further subtypes, for example, Haemophagocytic HS (HHS) will be found mainly in abdominal and Langerhans cell histiocytosis (LCH) externally. The distribution single–multiple–diffuse can be added, for example, for an easier distinction of DHS. We found that when multiple or diffuse lesions are seen in an internal organ with imaging or pathology, DHS is often present. It can also help for an easier distinction of HHS, which can be recognized by a mostly diffuse distribution in the tissues [30]. Specific organs can be added to identify specifically involved cell lineages.

In the majority of cases (BMD 82.2% and FCR 87.2%), only one body compartment seems to be involved by HS. It is possible that this is influenced by owners that often request that the disease is diagnosed with as little effort and cost as possible, and therefore, only one, most easily reached, compartment is examined, but it is also possible that HS not so often crosses boundaries of body compartments.

DHS [1] and HHS [30] are suspected to arise in the red pulp of the spleen or bone marrow, but interestingly, almost no cases with bone marrow involvement were found in our study, as well as in the literature. Involvement of the bone marrow was only reported with a higher percentage in two other/multi-breed studies [1,35], but frequently in combination with a bone lesion which also involves the bone marrow [1]. One study concluded that almost 10% of bone marrow neoplasms in dogs are HS [66] and that scatterplots can be useful to differentiate hemophagocytic disorders of the bone marrow [67], but further research is warranted.

This is the first report about the involvement of abdominal lymph nodes in BMD and FCR; only a variable percentage in two small multi-breed studies was reported before [56,68]. Involvement of the adrenal glands is reported only once before BMD [2] and also never before in FCR. Interestingly, our percentage is lower than in some other/multi-breed reports [1,3,21], while according to one study, 20.4% of HS metastases to the adrenal glands in dogs [69].

This study has the advantage of having a very large number of dogs of only two specific breeds, making it possible to find rare characteristics and compare frequencies between the two breeds. However, there are also limitations due to the fact that it was a retrospective study. Staging was not performed on all dogs and in a uniform way. Therefore, the data could have been erroneously influenced by the unequal methods of diagnosis for staging, i.e., by diagnosing localized HS when it was actually disseminated and, in general, underestimating the number of organs affected in each dog. The decision to perform a blood examination could have been influenced by the type of HS. DHS dogs had significantly more frequent blood examinations performed than LHS dogs, both in BMD and FCR, likely because of the systemic nature of the presenting clinical signs. Therefore, the reported frequencies should be interpreted with care.

Concerning the extremities in the FCR literature, the elbow is most often reported [4,29,31,70,71], while in our larger study, no significant difference in frequency was observed for the shoulder, elbow, and knee. In BMD, the knee was the most frequently affected joint.

## 5. Conclusions

With the help of a large sample of cases and an extensive diagnostic process used in this study, more insight has been obtained into the complex and aggressive disease process of HS in BMD and FCR. The new information provided in this study can aid the diagnostic process and allow for prompt treatment recommendations. Overall, it can be concluded that in BMD and FCR, even from the young age of 0.6 years old, presenting with general, non-specific malaise or with one or more of the above-mentioned signs, HS has to be included in the differential diagnosis, and further examination has to start quickly to exclude HS, because of the high prevalence and grave prognosis associated with HS.

## Figures and Tables

**Table 1:** Comparison of patient descriptives of FCR and BMD with HS.

Parameter	FCR	BMD	*p*-Value
Number of dogs	289	365	
Age (years; mean, range)	8.2 (0.6–13.7)	6.6 (0.6–12.2)	<0.001
Sex			0.981
Male (number, %)	142 (49.1%)	181 (49.6%)	
Female (number, %)	147 (50.9%)	184 (50.4%)	
Neutered (all sexes)	109 (37.7%)	189 (51.8%)	<0.001
Male	39 (27.6%)	75 (41.4%)	0.010
Female	70 (47.6%)	114 (61.9%)	0.013
HS-type			<0.001
Localized	175 (60.6%)	143 (39.2%)	
Disseminated	114 (39.4%)	222 (60.8%)	
TimeToDx (mean, range) ^a^	35.6 (0–500)	27.9 (0–377)	0.103
FUperiod (mean, range) ^b^	105.6 (0–2507)	102.5 (0–2570)	0.888

^a^: Period of time from first signs to diagnosis; ^b^: Follow-up period.

**Table 2:** Comparison of clinical signs in FCR and BMD with HS.

Parameter	FCR	BMD	*p*-Value
Number of dogs	289	365	
Pale mucous membranes	67 (23.3%)	151 (41.4%)	<0.001
Icterus	9 (3.1%)	26 (7.1%)	0.023
Pyrexia	35 (12.2%)	92 (25.3%)	<0.001
Lameness	114 (39.5%)	87 (23.8%)	<0.001
Front leg	89 (78.1%)	38 (43.7%)	<0.001
Hind leg	25 (21.9%)	44 (56.3%)	
Neurologic Symptoms	13 (4.5%)	14 (3.8%)	0.776
Ataxia	2	7	
Epilepsy	2	2	
Paresis/paralysis	10	10	
Weight loss	46 (15.9%)	132 (36.2%)	<0.001
Ascites	5 (1.7%)	15 (4.1%)	0.078
Anorexia	75 (26.0%)	207 (56.7%)	<0.001
Vomiting	19 (6.6%)	29 (7.9%)	0.504
Diarrhea	5 (1.7%)	10 (2.7%)	0.389
Polydipsia	21 (7.3%)	30 (8.2%)	0.652
Dyspnoea	19 (6.6%)	38 (10.4%)	0.080
Coughing	13 (4.5%)	41 (11.2%)	0.001
Lethargy	98 (33.9%)	215 (58.9%)	<0.001

**Table 3:** Blood results differentiated for breed (FCR vs. BMD) and type of HS (LHS vs. DHS).

Parameter	FCR	BMD	*p*-Value	LHS	DHS	*p*-Value
Anemia						
PCV < ref.value	58/78 (74.4%)	124/160 (77.0%)	0.650	46/67 (68.7%)	136/171 (79.5%)	0.075
PCV< 0.30 l/l	24/78 (30.8%)	63/160 (39.1%)	0.384	9/67 (13.4%)	78/171 (45.6%)	<0.001
Leukocytosis	15/44 (34.1%)	72/108 (66.7%)	<0.001	20/39 (51.3%)	67/113 (59.3%)	0.383
Thrombocytopenia	14/41 (34.1%)	52/94 (55.3%)	0.027	7/31 (22.6%)	59/104 (56.7%)	0.003
Elevated AP	34/43 (79.1%)	60/87 (69.0%)	0.316	21/33 (63.6%)	70/93 (75.2%)	0.200
Hypoalbuminemia	26/41 (63.4%)	61/94 (64.9%)	0.869	14/36 (38.9%)	65/99 (65.7%)	0.626
Hypercalcemia	0/23 (0%)	15/53 (28.3%)	<0.001	4/23 (17.4%)	11/53 (20.8%)	0.735
Abn. coagulation	4/5	8/17	0.179	1/3	11/19	0.427

**Table 4:** Comparison of tumor location of HS in FCR and BMD.

Parameter	FCR	BMD	*p*-Value
*Clinical parameters*	*n = 289*	*n = 365*	
External lymph nodes	71 (24.6%)	60 (16.4%)	0.010
Limb			<0.001
Front leg	102 (35.2%)	25 (6.8%)	
Shoulder	33	3	
Elbow	30	18	
Carpal joint	2	1	
Other	37	3	
Hind leg	31 (10.7%)	36 (9.8%)	
Knee	15	28	
Tarsal joint	2	1	
Other	16	7	
Eye	1 (0.3%)	6 (1.6%)	0.087
CNS ^a^	5 (1.7%)	3 (0.8%)	0.312
Soft tissue head/neck/trunk	48 (16.6%)	58 (15.9%)	0.804
*Abdominal organs (based on imaging, expl laporatomy, and p.m. examination)*	*n = 88*	*n = 185*	
Spleen			<0.001
Single mass	14 (15.9%)	10 (5.4%)	
Multiple masses	49 (55.6%)	49 (26.5%)	
Diffuse	1 (1.1%)	43 (23.2%)	
Diffuse + masses	1 (1.1%)	11 (5.9%)	
Liver			0.003
Single mass	2 (2.3%)	9 (4.9%)	
Multiple masses	29 (32.9%)	57 (30.8%)	
Diffuse	3 (3.4%)	37 (20.0%)	
Diffuse + masses	0	3 (1.6%)	
Lymph nodes			0.939
Abdominal	21 (23.9%)	45 (24.3%)	
Pelvic	4 (4.5%)	11 (5.9%)	
Abd + pelvic	0	4 (2.2%)	
Kidney			0.231
Unilateral	3 (3.4%)	6 (3.2%)	
Bilateral	7 (2.4%)	6 (1.6%)	
Gastrointestinal tract	2 (2.3%)	3 (1.6%)	0.169
*Thoracic organs (based on imaging and p.m. examination)*	*n = 73*	*n = 167*	
Lungs			0.058
Single mass	10 (13.7%)	33 (19.8%)	
Multiple masses	15 (20.5%)	52 (31.1%)	
Thor. lymph nodes	15 (20.5%)	40 (23.9%)	0.564
Heart			0.572
Pericard	2 (2.7%)	2 (1.2%)	
Myocard	1 (1.4%)	1 (0.6%)	

^a^: brain and spinal cord.

## Data Availability

Not applicable.
